# Fluorescent Staining Using Blankophor for the Diagnosis of Sporotrichosis on Fresh Biopsies

**DOI:** 10.1007/s11046-025-01041-6

**Published:** 2026-01-06

**Authors:** Regielly Caroline Raimundo Cognialli, Marisol Dominguez Muro, Vânia Aparecida Vicente, Jacques F. Meis, Eelco F. J. Meijer, Flávio Queiroz-Telles

**Affiliations:** 1 Hospital de Clínicas, Federal University of Paraná, Curitiba, Brazil; 2 Postgraduate Program in Microbiology, Parasitology and Pathology, Federal University of Paraná, Curitiba, Brazil; 3 Department of Basic Pathology, Federal University of Paraná, Curitiba, Brazil; 4 Institute of Translational Research, Cologne Excellence Cluster On Cellular Stress Responses in Aging-Associated Diseases (CECAD), University of Cologne, Cologne, Germany; 5 Radboudumc-CWZ Center of Expertise for Mycology, Nijmegen, The Netherlands; 6 Department of Medical Microbiology and Immunology, Canisius-Wilhelmina Hospital (CWZ)/Dicoon, Nijmegen, The Netherlands; 7 Department of Public Health, Hospital de Clínicas, Federal University of Paraná, Curitiba, Brazil

**Keywords:** Sporotrichosis, Sporothrix brasiliensis, Diagnosis, Biopsy, Blankophor

## Abstract

Sporotrichosis is an implantation mycosis with a high incidence in Brazil. Diagnosing human sporotrichosis poses significant challenges, which can lead to increased morbidity and prolonged treatment duration. Direct examination of fresh biopsies using Blankophor represents a valuable tool for rapid diagnosis, offering high sensitivity.

## Short Communication

Sporotrichosis is caused by thermodimorphic fungi of the *Sporothrix* genus [1]. In recent decades, a new species, *S. brasiliensis*, has emerged in Brazil, leading to a large outbreak that has spread to other countries [2–5]. The case definition of sporotrichosis classifies cases as possible, probable, or proven, based on epidemiological, clinical, and laboratory diagnostic criteria [6]. Most cases are classified as probable due to challenges in performing fungal cultures and awaiting results, prompting the initiation of empirical treatment [6]. Additionally, atypical manifestations are often poorly recognized by clinicians, necessitating thorough differential diagnosis [7].

Fungal culture remains the gold standard for diagnosis of sporotrichosis; however, its sensitivity varies depending on factors such as fungal burden, prior treatment, potential bacterial contamination, and the type of specimen collected [8, 9]. For biopsies, the average growth time in culture is 13 days, which is shorter for pus specimens (6 days) [6]. Conventional direct examination using 10–40% potassium hydroxide (KOH) has a sensitivity of only 1–2% [8]. Histopathological studies show sensitivity ranging from 18–35%, depending on the technique used [9]. Microscopic analysis has low sensitivity, mainly due to the scarcity of yeast structures in the specimen [8, 9]. Serological methods have been validated and are promising, particularly ELISA and lateral flow assays; however, commercial kits are not yet available [10]. Molecular tests to detect *Sporothrix* DNA directly in clinical samples have also been studied, but commercial kits for these tests are not yet available either [8]. Nevertheless, for species identification from fungal isolates, some molecular techniques are employed, such as calmodulin gene sequencing and genotyping [11, 12].

Fluorescent staining of fungi is a useful technique with high sensitivity for clinical specimens in histopathological and direct examinations, but it is not routinely used in clinical laboratories [8, 13, 14]. Various solutions can be used, such as optical brighteners like Calcofluor White and Blankophor which bind to chitin and cellulose [13]. Blankophor {4,4′-bis[(4-anilino-subst.1,3,5-triazin-2-yl)amino]stilben-2,2′-disulfonic acid} is diluted in 15–20% potassium hydroxide, which facilitates the digestion of clinical specimens [14]. After preparing slides with fresh specimens, the examination is conducted using a fluorescent microscope with an excitation wavelength below 400 nm and a barrier filter at 420 nm [14]. The fluorescence intensity can increase over time; it is often stronger after 24 h, but fungal structures can usually be visualized within 15 min of preparation [14].

We conducted direct examinations using 40% KOH and Blankophor on eight fresh tissue biopsies—seven from humans and one from a dog. All patients presented clinical suspicion of probable zoonotic sporotrichosis, in accordance with the Brazilian Health Surveillance Guidelines, exhibiting compatible lesions and a history of epidemiological contact with infected cats [6]. All skin biopsies were collected under sterile conditions using a 2–3 mm punch technique, immediately placed in sterile containers containing saline solution, transported to the laboratory without delay, and processed within 6 h of collection. Among the human cases (n = 7), three exhibited the fixed cutaneous form and four displayed the lymphocutaneous form of infection. The anatomical distribution of human biopsy sites included: three from lower limbs (42.9%), three from upper limbs (42.9%), and one from the abdomen (14.2%). Human biopsy specimens measured 2–3 mm in diameter. The canine case (n = 1) involved a 2 mm diameter biopsy collected from a nasal bridge lesion.

Following our standard protocol, biopsies were aseptically transferred to a sterile Petri dish in a Biological Safety Cabinet, immersed in sterile water containing gentamicin solution, and excess fluid was removed. The tissue was vertically minced into small fragments using a sterile scalpel and cultured on Sabouraud dextrose agar (HiMedia, Mumbai, India), Mycosel agar (Becton Dickinson, Ontario, Canada), and Brain Heart Infusion agar (HiMedia, Mumbai, India) at 30 °C. For microscopic analysis, two slides were prepared per biopsy: one with 40% KOH and another with Blankophor. To prepare the slides, biopsy fragments were placed on a microscope slide, excess liquid was removed, one drop of 40% KOH or Blankophor was added, and the preparation was then covered with a coverslip. The slides were labeled with random numbers to ensure that the mycologists remained blinded to the study. All slides were kept in a moist chamber to prevent drying. After two hours, slides were examined by an experienced mycologist (> 10 years of experience); KOH slides were observed under an Olympus CX31 microscope (Japan), while Blankophor slides were evaluated using a Zeiss Axioskop 2 mot plus microscope (Germany) equipped with a DAPI (4',6-diamidino-2-phenylindole) filter (excitation: 358 nm; emission: 461 nm).

**Fig. 1:**
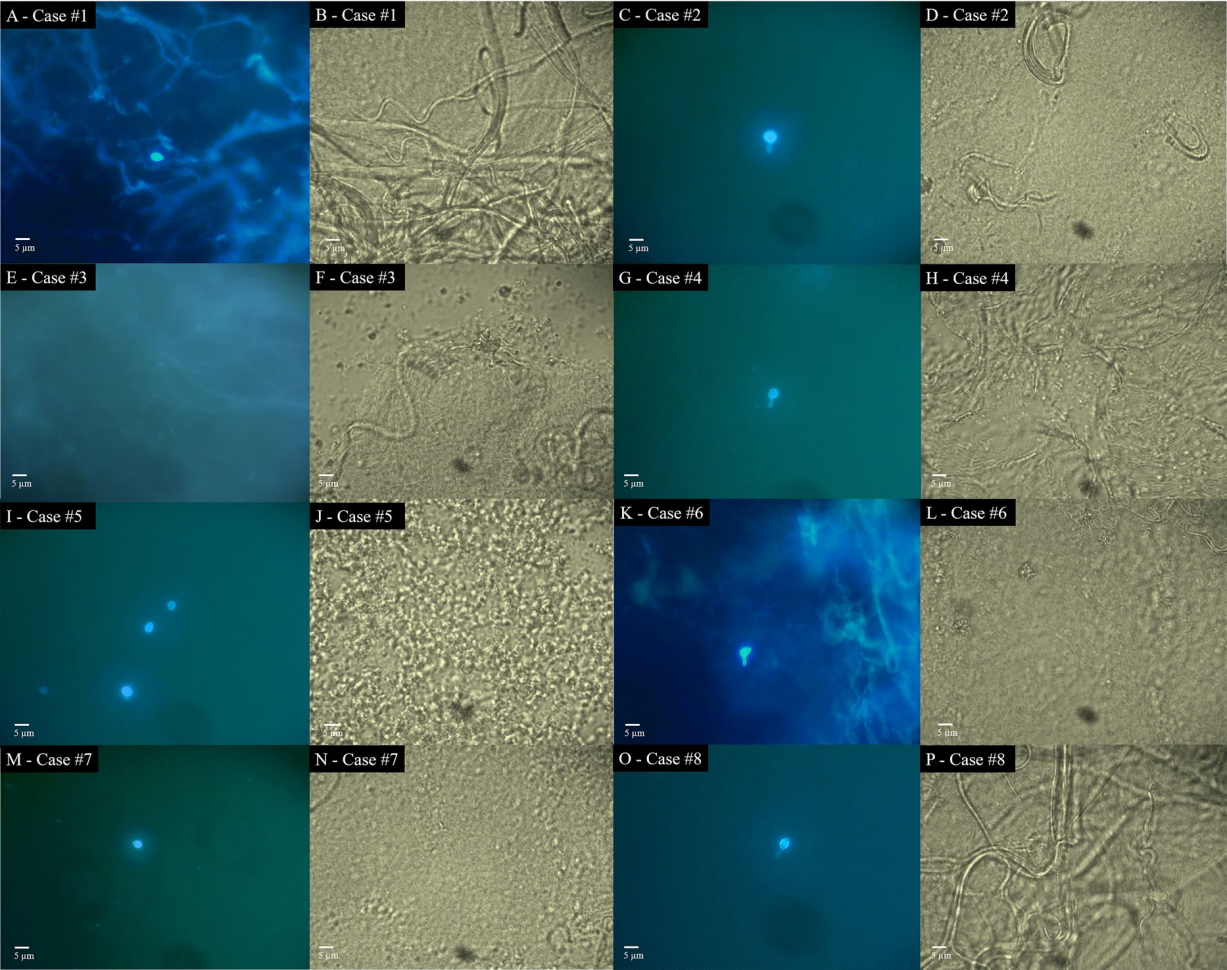
Comparative direct microscopic examination of fresh biopsy specimens using Blankophor and 40% KOH across all cases. * Legend*: **A** Blankophor fluorescent stain from Case #1 showing yeast structure morphologically consistent with *Sporothrix* spp. **B** Corresponding 40% KOH preparation from Case#1 shows no detectable fungal elements. **C** Blankophor fluorescent stain from Case #2 showing yeast structure morphologically consistent with *Sporothrix* spp. **D** Corresponding 40% KOH preparation from Case#2 shows no detectable fungal elements. **E** Blankophor fluorescent stain from Case #3 shows absence of fungal structures. **F** Corresponding 40% KOH preparation from Case#3 shows no detectable fungal elements. **G** Blankophor fluorescent stain from Case #4 showing yeast structure morphologically consistent with *Sporothrix* spp. **H** Corresponding 40% KOH preparation from Case#4 shows no detectable fungal elements. **I** Blankophor fluorescent stain from Case #5 showing multiple yeast structures morphologically consistent with *Sporothrix* spp. **J** Corresponding 40% KOH preparation from Case#5 shows no detectable fungal elements. **K** Blankophor fluorescent stain from Case #6 showing yeast structure morphologically consistent with *Sporothrix* spp. **L** Corresponding 40% KOH preparation from Case#6 shows no detectable fungal elements. **M** Blankophor fluorescent stain from Case #7 showing yeast structure morphologically consistent with *Sporothrix* spp. (N) Corresponding 40% KOH preparation from Case#7 shows no detectable fungal elements. **O** Blankophor fluorescent stain from Case #8 showing yeast structure morphologically consistent with *Sporothrix* spp. **P** Corresponding 40% KOH preparation from Case#8 shows no detectable fungal elements. Scale bar = 5 μm (consistent across all panels)

Figure 1 summarizes the findings observed in direct examination for each case using Blankophor and 40% KOH (Fig. 1). Fungal structures were not visualized in any of these cases using conventional direct examination with 40% KOH, likely due to the low fungal burden and interference from artifacts and tissue fibers. However, when Blankophor was employed, scarce yeast cells were observed in six cases. In one case (Case #5), a higher number of yeast cells was observed, likely due to the patient’s advanced HIV infection. The yeast cells were oval to round, with some elongated and "cigar-shaped”, often with “pipe stem” budding, measuring approximately 4–6 µm. The only case negative in the direct examination with Blankophor was from a human patient presenting with the fixed cutaneous form (Case #3). *Sporothrix* was successfully isolated in culture from all eight specimens in all culture media employed. The fungal isolates were deposited at Micobiological Collections of the Paraná Network–Taxonline (CMRP/Taxonline: https://www.cmrp-taxonline.com). Fungal isolates were identified by partial calmodulin gene sequencing in seven cases as *S. brasiliensis* and in one case as *S. schenckii*. The generated sequences were deposited in GenBank. Table 1 summarizes the clinical features, along with the results from direct examination, culture, and the corresponding accession numbers from CMRP and GenBank. The sensitivity of Blankophor for detecting sporotrichosis in fresh biopsies was 87%.

**Table 1:** Clinical characteristics, mycological findings (KOH and Blankophor direct examination), and strain accession numbers (CMRP/GenBank) of sporotrichosis cases included in the study

Case ID	Source	Clinical form	Biopsy site	Direct examination with 40% KOH	Direct examination with Blankophor	Culture	CMRP / GenBank accession numbers
#1	Human	LC	Left upper limb	Negative	Positive	*S. brasiliensis*	CMRP6648 / PV740341
#2	Canine	FC	Nasal bridge	Negative	Positive	*S. brasiliensis*	CMRP6863 /PV740333
#3	Human	FC	Abdomen	Negative	Negative	*S. brasiliensis*	CMRP6647 / PV740348
#4	Human	LC	Left upper limb	Negative	Positive	*S. brasiliensis*	CMRP6624 /PV740339
#5	Human	FC	Left lower limb	Negative	Positive	*S. brasiliensis*	CMRP6191 /PV40346
#6	Human	LC	Right lower limb	Negative	Positive	*S. schenckii*	CMRP7272 / PX000723
#7	Human	FC	Left lower limb	Negative	Positive	*S. brasiliensis*	CMRP7274 / PX000724
#8	Human	LC	Left upper limb	Negative	Positive	*S. brasiliensis*	CMRP7275 / PX000725

FC: fixed cutaneous; LC: lymphocutaneous

The fluorescent brightener Blankophor, which binds specifically to fungal cell wall components, is a fast and reliable staining technique to detect fungi in clinical specimens as early as 1951 [15, 16]. Fluorescent staining for the diagnosis of sporotrichosis has previously been described for histopathological examination, demonstrating a high sensitivity of 65% [17, 18]. However, the use of Blankophor in fresh tissue biopsies offers a rapid turnaround time (less than 24 h), is cost-effective, involves a simple technique, and represents a valuable alternative for endemic regions, improving laboratory diagnosis. Waiting for culture results can lead to delayed diagnosis, increasing morbidity and prolonging treatment duration.

The primary limitation of our study is the small sample size, comprising only eight skin biopsies. Additionally, the study was conducted in a specialized mycology laboratory located in an endemic region for sporotrichosis in Brazil, which may have influenced the high sensitivity observed in our results. While Blankophor fluorescent staining shows promise for diagnosing sporotrichosis in fresh biopsies, this method requires experienced personnel due to the typically low fungal burden and small yeast cell diameter. Although direct examination is not always conclusive for diagnosing sporotrichosis, it can guide the diagnostic process, particularly in cases with clinical suspicion and the need for differential diagnosis. Future studies with larger sample sizes should validate these preliminary findings and include comparative evaluations with other fluorescent stains, such as Calcofluor white, to better establish the method's diagnostic performance characteristics.
